# Subjective health complaints in patients with lumbar radicular pain and disc herniation are associated with a sex - *OPRM1 A118G* polymorphism interaction: a prospective 1-year observational study

**DOI:** 10.1186/1471-2474-15-161

**Published:** 2014-05-18

**Authors:** Eivind Hasvik, Elina Iordanova Schistad, Lars Grøvle, Anne Julsrud Haugen, Cecilie Røe, Johannes Gjerstad

**Affiliations:** 1 National Institute of Occupational Health, Oslo, Norway; 2 Department of Physiotherapy, Østfold Hospital Trust, Fredrikstad, Norway; 3 Department of Physical Medicine and Rehabilitation, Oslo University Hospital, Ullevål, Norway; 4 Department of Rheumatology, Østfold Hospital Trust, Fredrikstad, Norway; 5 Department of Biosciences, University of Oslo, Oslo, Norway

**Keywords:** μ-opioid receptor 1, Sex, Subjective health complaints, Sciatica, Radiculopathy, *OPRM1 A118G*, rs1799971

## Abstract

**Background:**

Earlier observations show that development of persistent pain may be associated with the genetic variability in the gene encoding for the μ-opioid receptor 1, the *OPRM1 A118G* (rs1799971). The aim of this study was to investigate the association between *OPRM1* genotype and subjective health complaints in patients with radicular pain and disc herniation.

**Methods:**

A prospective, 1-year observational study was conducted at a hospital back clinic, including 118 Caucasian patients with lumbar radicular pain and MRI confirmed disc herniation. Single nucleotide polymorphism genotyping regarding the *OPRM1 A118G* was performed. The data of individuals with AA versus AG or GG were analysed separately by linear mixed models. The Subjective Health Complaints Inventory (0-81) including 27 common complaints experienced the previous month on a scale from *not at all* (0) to *severe* (3) was used as outcome. Pain, prior duration of leg pain, age, smoking status, and lumbar disc surgery were considered as covariates.

**Results:**

In total 23 of 118 patients were carriers of the *OPRM1* G-allele. All patients except female carriers of the G-allele reported a decrease in pain from baseline to 1 year. Female carriers of the G-allele reported significantly higher subjective health complaints score during the study time span than male carriers of the G-allele when controlling for pain and pain duration.

**Conclusion:**

The present data indicate that, when controlling for pain intensity and duration, subjective health complaints are associated with a sex - *OPRM1 A118G* polymorphism interaction in patients with radicular pain.

## Background

Patients with non-specific low back pain or lumbar radicular pain often report additional subjective health complaints [1-4]. In the literature such health complaints have also been referred to as multiple physical symptoms, functional somatic syndrome, medically unexplained symptoms or somatisation [5-8], and include report of headaches, pain, dyspnoea, gastrointestinal discomfort, anxiety and sadness [9]. In patients with lumbar radicular pain, previous work has shown that subjective health complaints may be associated with the severity of pain-related disability [2] and unfavourable outcome at 1 and 2 years follow-up [10].

The single nucleotide polymorphism (SNP) in the human gene coding for the μ-opioid receptor 1, the *OPRM1 A118G* (rs1799971), may affect nociceptive signalling. For example, carriers of the G-allele seem to have less pronounced effects of opioids [11-15], increased pressure pain thresholds [16] and reduced cortical responses to nociceptive olfactory CO_2_ stimulation, indicating decreased nociceptive activation [17]. However, the G-allele has been associated with higher pain ratings and amount of self-administered intravenous morphine in women after caesarean section [18,19]. Moreover, female carriers of the G-allele suffering from lumbar radicular pain and disc herniation reported significantly higher pain levels than males with the same genotype [20].

Of relevance to comorbid symptoms, the *OPRM1* polymorphism has been associated with other physiological processes such as immune and inflammatory function [21,22], stress response [23,24], substance abuse [25], social attachment [26], social sensitivity to rejection [27], and reward mechanisms [28,29]. However, there is a scarcity of comparable genetic work done on the report of subjective health complaints. Hence, the aim of the present study was to investigate how this genetic variant affects such comorbid symptoms in patients with lumbar disc herniation and radicular pain. Previous data have demonstrated sex differences between male and female G-allele carriers regarding pain [20]. Our hypothesis was therefore that the effect of the *OPRM1* G-allele could be different in men and women regarding the report of subjective health complaints.

## Methods

### Patients, setting and procedures

This study was part of a prospective, 1 year observational study on patients with suspected lumbar radicular pain and disc herniation who were referred to a back clinic at Oslo University Hospital, Ullevål (OUS), Norway, during 2007 to 2009. Patients were invited to participate by the clinic staff. Inclusion criteria were age 18-60 years, lumbar radicular leg pain with a corresponding lumbar disc herniation at the relevant side and level confirmed by magnetic resonance imaging, and positive straight leg raise test (defined as radiating pain below the knee, within 60° elevation). Exclusion criteria were lumbar spinal stenosis, previous disc surgery at the same level, lumbar spine fusion at any level, generalised musculoskeletal pain, inflammatory rheumatic disease, diabetic polyneuropathy, cardiovascular disease, cancer, psychiatric disease, neurological disease, alcohol or drug abuse, completion of another surgery within 1 month, pregnancy, non-detectable genotype, non-European-Caucasian ethnicity or poor Norwegian language. Patients received a standard clinical examination and answered a comprehensive questionnaire at baseline and at 1 year. Blood samples were taken at inclusion.

Treatment followed practice as usual, without any pre-allocation or randomisation. The conservative management options comprised a brief cognitive intervention, activity guidance during the acute phase, and physiotherapy. The choice of conservative and/or surgical treatment was made at the discretion of each physician/surgeon. All participants received written information and signed an informed consent form. The study was approved by the Norwegian Regional Committee for Medical Research Ethics and the Norwegian Social Science Data Services.

### Measurements

The number and severity of health complaints was measured by the Subjective Health Complaints Inventory (SHC) [9]. This questionnaire consists of 29 common somatic and psychological complaints, such as pain (6 items), headache, dyspnoea, gastrointestinal discomfort, anxiety and sadness (see Additional file 1 for full overview of questionnaire items). Participants grade the intensity of each complaint experienced during the previous month on a 0-3 scale with the categories; *not at all*, *a little*, *some*, and *severe*. The SHC score is calculated by summation of item scores. The items *leg pain during physical activity* and *low back pain* are closely related to the patient’s present pain complaint and consequently excluded in all analyses, making the total possible score 81. Data on SHC prevalence in the Norwegian general population [30] as well as comparisons with clinical samples have been published [2,4,31]. Pain severity was assessed by a 10-point numeric scale with endpoints *no pain* and *worst possible pain*, indicating pain in activity during the last week, as used previously [20]. Patients also reported duration of their leg pain prior to baseline. At inclusion the socio-demographic variables age, sex and smoking status were recorded.

### Genotyping

Genotyping was performed after finished patient sampling. Genomic DNA was extracted from whole blood cells using FlexiGene DNA isolation kit (Qiagen), and single nucleotide polymorphism (SNP) genotyping was performed using predesigned TaqMan SNP genotyping assays (Applied Biosystems). Approximately 10 ng of genomic DNA was amplified in 5 μl reaction mixture using a 384-well plate containing 1x TaqMan genotyping master mix (Applied Biosystems) and 1x assay mix. The latter contained the respective primers and probes. Probes were labelled with the TaqMan reporter dyes Fam™ and Vic® to distinguish between the *OPRM1* alleles, A or G. After initial denaturation and enzyme activation at 95°C for 10 min, the reaction mixture was subjected to 60 cycles of 95°C for 15 seconds and 60°C for 1 min. The reactions were performed on an ABI 7900HT sequence detection system. Negative controls containing water instead of DNA were included in every run. Genotypes were determined using the SDS 2.2 software (Applied Biosystems). Approximately 10% of the samples were re-genotyped, and the concordance rate was 100%.

### Analyses and statistics

Genotypes were dichotomised into AA carriers (participants with homozygous A-alleles) or *G carriers (participants with heterozygous A/G-alleles or homozygous G-alleles) because only one patient was homozygous GG. Analyses were performed separately for female *G versus male *G carriers, female AA versus female *G carriers, male AA versus male *G carriers, as well as female AA versus male AA carriers, using a linear mixed effects model approach. The outcome variable was SHC during the study time span. Both baseline and 1-year scores were included in the model. This was done to examine change over time by testing interactions between SHC and time. Pain, as measured at baseline and 1 year, duration of leg pain prior to inclusion, age, smoking status, and lumbar disc surgery during the follow-up were tested in univariate analyses. Multivariate analyses were built by including variables from the univariate analyses with p < 0.1. The baseline model consisted of the fixed factors shown in Table 1. Subsequent models tested random intercept, and random slope for pain intensity. The decision to test for random effects of pain was based on previous work showing genotype differences in pain intensity over time [20]. Subject-specific random factors and covariance structure was based on a *best fit* approach, assessed by the small-sample version of Akaike’s information criteria (AICc) [32]. The final model random factors included random intercept, and random slope for pain intensity. Scaled identity covariance structure for the random factors was used. Estimation was done by maximum likelihood. No centring or imputation was performed. Analyses were assessed for adherence to linear mixed effect model assumptions. The four comparisons were corrected with the Bonferroni method. A p value < 0.0125 (0.05/4) was chosen as the significance level. Analyses were done using SPSS version 21.0 (IBM Corp., Armonk, NY).

**Table 1:** **Parameter estimates for predictors in the final linear mixed-effects regression model, with SHC during the study time span**^**† **^**as outcome (*n*** **= 23)**

	***b***	**SE**	**95% CI**	**p**
**Estimates of fixed effects**				
Female *G carrier (male *G)	6.59	2.37	1.2, 11.37	0.008
Pain score (0-10)^†^	0.92	0.39	0.12, 1.72	0.025
Pain duration (weeks)	0.14	0.05	0.04, 0.25	0.010
Time 1 year (baseline)	1.91	1.13	-0.42, 4.24	0.103
**Estimates of covariance parameters**				
Residual	9.19	3.04	4.80, 17.59	0.003
Random effects	1.75	0.65	0.85, 3.63	0.007

Reference category or scale in brackets. †Both baseline and 1-year scores were included in the analysis.

## Results

**Figure 1:**
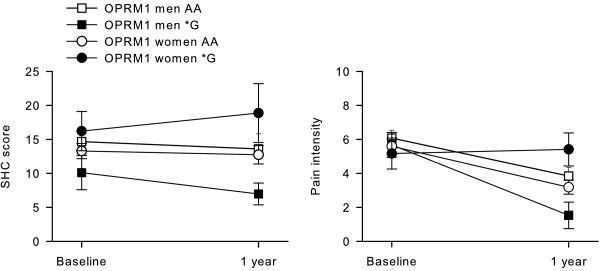
Subjective health complaints and pain scores (mean values ± SEM) according to sex and genotype.

In total, 118 patients were included in the study of whom 112 (95%) completed the 1-year follow-up. 23 patients were carriers of the *OPRM1* G-allele, corresponding to a G-allele frequency of 10.2% in total (14% among males, 7.3% among females). One woman was homozygous GG. Patient characteristics according to sex and genotype are presented in Table 2. All patients except the female *G-carriers reported a decrease in pain from baseline to 1 year. Figure 1 illustrates the SHC and pain scores at baseline and 1 year according to sex and genotype.

**Table 2:** Patient characteristics according to sex and genotype

	***OPRM1 *** **AA genotype**		***OPRM1 *** *G genotype	
	**Men**	**Women**	**Men**	**Women**
N	36	59	14	9
Age	42.9 (10.3)	39.6 (9.5)	42.4 (10.8)	43.2 (8.9)
SHC baseline	14.67 (9.63)	13.29 (8.55)	10.10 (9.38)	16.22 (8.67)
SHC 1 year	13.61 (12.50)	12.73 (10.02)	6.97 (6.01)	18.88 (12.23)
Pain baseline	6.07 (2.68)	5.66 (2.66)	5.68 (3.14)	5.17 (2.50)
Pain 1 year	3.84 (3.12)	3.12 (2.90)	1.53 (2.65)	5.41 (2.32)
Duration (weeks)	22.6 (18.9)	33.3 (30.9)	17.5 (14.5)	40.7 (37.8)
Smoker	50%	27%	36%	44%
Surgery	44%	37%	36%	33%

Values presented as means with standard deviation (SD).

The results from the final linear mixed model, including pain intensity and duration, showed that female *G carriers had significantly higher SHC score than male *G carriers, F (1, 44) = 7.73, p = 0.008. This effect of sex had a clear influence on SHC (*b =* 6.59, 95% CI 1.2, 11.37), whereas the effect of pain (*b* = 0.92, 95% CI 0.12, 1.72, p = 0.025) and prior pain duration (*b* = 0.14, 95% CI 0.04, 0.025, p = 0.01) was less pronounced. The subject-specific random factors had a significant effect in the final model (*b* = 1.75, 95% CI 0.85, 3.63, p = 0.007). When including SHC x time interaction the overall model fit was significantly reduced and this interaction was therefore not included in the final model. The final model and estimates of covariance parameters is presented in Table 1.

The SHC score did not differ significantly between female AA and *G carriers, F (1, 63) = 1.63, p = 0.206, between male AA and *G carriers, F (1, 48) = 2.48, p = 0.122 or between female and male AA carriers, F (1, 88) = 0.63, p = 0.43.

## Discussion

The present study shows that multiple subjective health complaints (SHC) are associated with the *OPRM1* polymorphism. During the study time span female *G carriers reported significantly higher SHC scores than male *G carriers. This was true also when controlling for pain intensity and prior duration of pain. Hence, the present data demonstrate that SHC may be a trait that in itself is linked to the *OPRM1* genotype. Moreover, our results show that SHC in patients with lumbar radicular pain and disc herniation are associated with a specific sex - *OPRM1* polymorphism interaction. Previous data demonstrate an increase in SHC in patients with longer duration of radicular pain, indicating SHC to be secondary to pain and disability [2]. Our analyses however, suggest that the increased SHC might be more than just a phenomenon secondary to pain. No difference was found between female AA and *G carriers, male AA and *G carriers or female and male carriers of the AA genotype.

The sex - *OPRM1* polymorphism interaction observed in the present study clearly support earlier findings. For example, it has been reported that μ-opioid receptor binding potential may be higher with increasing age and in women [33]. Moreover, in one human experimental study, region-specific differences in *OPRM1* levels between individuals with AA and G alleles have been demonstrated [34]. Additionally, the *OPRM1* genotype may influence stress responses, immune and pro-inflammatory responses [21,35-37], and reactivity to social rejection [27]. Also, acute and chronic stress has been suggested to affect available μ-opioid receptor pools in GABAergic interneurons differentially in female and male rats [38]. Exactly how this happens is not fully understood.

It is, however, now well established that the *OPRM1* polymorphism leads to a substitution of the amino acid asparagine (N) to aspartic acid (D) at position 40 of the extracellular receptor region. This affects the glycosylation site of the receptor, which is important for cellular processes such as receptor folding, sorting, expression and ligand binding [39]. The level and type of glycosylation are different between female and male mice [40-42], which may explain the sex-differences [43]. Hence, we think the OPRM1 polymorphism in a sex-specific manner also may affect supraspinal neuronal signaling in humans.

The SHC Inventory captures similar symptoms as described in phenomenon such as medically unexplained symptoms or somatisation [5,7,9]. Somatisation symptoms, when not adequately explained by organic findings, have been conceptualised as either I) somatic manifestation of psychological distress, II) or as somatic distress and the experience of medically unexplained symptoms without a strong assumption about causality, or III) as somatic syndromes characterised by specific clusters of somatic symptoms [44,45]. The present study cannot differentiate between these concepts, but gives support to the argument that we should avoid simple psychological explanations of these physical complaints [8]. Previous findings have shown a similar association between a five-item questionnaire pertaining to unexplained somatic symptoms in the general population and genes relevant for the neuroendocrine system [46]. Taken together, genetically mediated variance of these complaints suggests that physiological processes influenced by genotype may be explanatory in some individuals’ symptom phenotype.

Duration of pain prior to baseline was an independent predictor for SHC in the present study. The female *G carriers had on average 23 weeks longer duration than male *G carriers. It is possible that duration of pain influenced the progress in SHC and pain scores - and that with longer durations also men would report increasing subjective health complaints. It has been shown in a similar cohort with lumbar radicular pain patients that those who report their pain as unchanged or worse, also report subjective health complaints as significantly elevated [2]. It is also possible that female *G carriers seek medical help at a later stage. However, female AA carriers had over 10 weeks longer duration than male AA carriers, without differing SHC scores. Moreover, male *G carriers had a shorter pain duration before inclusion and similar pain intensity, but still reported on average a faster recovery of their pain.

Previous studies have reported that individuals with one or more G-alleles have a reduced analgesic response to opioids. Thus, our lack of data on medication may be a potential confounder [15,47]. Moreover the inherent uncertainty in interpretation of genetic association studies and the low numbers of *G carriers emphasises the need for replication of our findings. Importantly, the frequency of G-variant carriers in the general population is low, 10.4% among Caucasians has been reported [12]. The present finding can therefore only explain some of the variance of subjective health complaints in total.

## Conclusion

The present data indicate that SHC in patients with radicular pain and disc herniation may be influenced by a polymorphism in the *OPRM1* gene in a sex-specific manner. We conclude that SHC may be a trait that in itself is linked to the *OPRM1* genotype.

## Abbreviations

SNP: Single nucleotide polymorphism; *OPRM1*: μ-opioid receptor 1; SHC: Subjective health complaints.

## Competing interests

The authors declare that they have no competing interests. No support was received from commercial sources.

## Authors’ contributions

EH, EIS, CR and JG participated in the research design. EIS, CR, JG carried out the data collection. EH, LG, AJH and JG carried out the data analysis and drafting. All authors read and approved the final manuscript.

## Pre-publication history

The pre-publication history for this paper can be accessed here:

http://www.biomedcentral.com/1471-2474/15/161/prepub

## Supplementary Material

Additional file 1 English version of the Subjective Health Complaints Inventory. Click here for file
