# Supplementary material for: Subjective health complaints in patients with lumbar radicular pain and disc herniation are associated with a sex - OPRM1 A118G polymorphism interaction: a prospective 1-year observational study
Source: BMC Musculoskelet Disord. 2014 May 18;15:161. doi: 10.1186/1471-2474-15-161 (PMC4038376; doi:10.1186/1471-2474-15-161)
Supplement: Additional file 1 — English version of the Subjective Health Complaints Inventory. [file 1471-2474-15-161-S1.pdf]

Additional file 1
English version of the Subjective Health Complaints Inventory.
As presented in Eriksen HR, Ihlebaek C, Ursin H. A scoring system for subjective health complaints (SHC). Scand J Public Health. 1999 Mar 1;27(1):63–72.

Ordinary health problems and complaints last month (circle the correct number)	Not at all	A little	Some	Serious	Number of days
1. Cold, flu	0	1	2	3	....
2. Coughing	0	1	2	3	....
3. Shoulder pain	0	1	2	3	....
4. Neck pain	0	1	2	3	....
5. Upper back pain	0	1	2	3	....
6. Arm pain	0	1	2	3	....
7. Headache	0	1	2	3	....
8. Low back pain *	0	1	2	3	....
9. Leg pain during physical activity *	0	1	2	3	....
10. Migraine	0	1	2	3	....
11. Anxiety	0	1	2	3	....
12. Sadness/depression	0	1	2	3	....
13. Sleep problems	0	1	2	3	....
14. Tiredness	0	1	2	3	....
15. Extra heartbeats	0	1	2	3	....
16. Heat flushes	0	1	2	3	....
17. Dizziness	0	1	2	3	....
18. Stomach discomfort	0	1	2	3	....
19. Heartburn	0	1	2	3	....
20. Ulcer/non-ulcer dyspepsia	0	1	2	3	....
21. Stomach pain	0	1	2	3	....
22. Gas discomfort	0	1	2	3	....
23. Diarrhoea	0	1	2	3	....
24. Obstipation	0	1	2	3	....
25. Asthma	0	1	2	3	....
26. Breathing difficulties	0	1	2	3	....
27. Allergies	0	1	2	3	....
28. Eczema	0	1	2	3	....
29. Chest pain	0	1	2	3	....

* The items *8. Low back pain* and *9. Leg pain during physical activity* were excluded in all analyses.
